# Comparison of TaqMan, KASP and rhAmp SNP genotyping platforms in hexaploid wheat

**DOI:** 10.1371/journal.pone.0217222

**Published:** 2019-05-22

**Authors:** Habtamu Ayalew, Pak Wah Tsang, Chenggen Chu, Junzhou Wang, Shuyu Liu, Caifu Chen, Xue-Feng Ma

**Affiliations:** 1 Noble Research Institute, Ardmore, Oklahoma, United States of America; 2 Center of Excellence for Research and Development, Integrated DNA Technologies, Redwood City, California, United States of America; 3 Texas A&M AgriLife Research, Amarillo, Texas, United States of America; USDA, UNITED STATES

## Abstract

Advances in high-throughput genotyping enable the generation of genome-scale data much more easily and at lower cost than ever before. However, small-scale and cost-effective high-throughput single-nucleotide polymorphism (SNP) genotyping technologies are still under development. In this study, we compared the performances of TaqMan, KASP and rhAmp SNP genotyping platforms in terms of their assay design flexibility, assay design success rate, allele call rate and quality, ease of experiment run and cost per sample. Fifty SNP markers linked to genes governing various agronomic traits of wheat were chosen to design SNP assays. Design success rates were 39/50, 49/50, and 49/50 for TaqMan, KASP, and rhAmp, respectively, and 30 SNP assays were manufactured for genotyping comparisons across the three platforms. rhAmp showed 97% of samples amplified while TaqMan and KASP showed 93% and 93.5% of amplifications, respectively. Allele call quality of rhAmp was 97%, while it was 98% for both TaqMan and KASP. rhAmp and KASP showed significantly better (p < 0.001) allele discrimination than TaqMan; however, TaqMan showed the most compact cluster. Based on the current market, rhAmp was the least expensive technology followed by KASP. In conclusion, rhAmp provides a reliable and cost-effective option for targeted genotyping and marker-assisted selection in crop genetic improvement.

## Introduction

Plant breeding is one of the applied research areas presumed to benefit greatly from the current advances in molecular marker technologies. Genome-scale sequencing technologies such as genotyping by sequencing (GBS) enable the generation of millions of single-nucleotide polymorphisms (SNPs) that can be used in genome-wide association studies (GWAS) to identify genes or quantitative trait loci (QTL) governing traits of interest [1–3]. Parallel developments in phenomics and statistical genetics enabled the identification of SNPs that are associated with desirable plant traits [4–7]. SNP markers are becoming preferred among breeders and molecular biologists because of their low-cost, high-genomic abundance, locus specificity, co-dominant inheritance, amenability to high-throughput genotyping, and relatively low genotyping error rates [8–10].

Genome-wide marker data have been used to identify many genes and QTL in wheat. The current challenge, however, is applying the identified markers in marker-assisted selection (MAS). Limited option of flexible and affordable small-scale high-throughput SNP genotyping has restricted MAS. Converting identified SNP regions into gel-based genotyping systems was reported as an option for small-scale genotyping, but it is obviously expensive and time-consuming [11, 12]. Genome-scale genotyping platforms are highly expensive and less flexible to use as a routine genotyping tool for targeted SNP assays [1, 10]. As a result, there has been a continuous effort to develop small-scale genotyping platforms. Small-scale SNP genotyping is also of prime importance for fingerprinting and quality control in crop breeding [13, 14].

Concerted research effort has developed small-scale high-throughput SNP genotyping platforms, including TaqMan and Kompetitive Allele Specific PCR (KASP) [14–16]. TaqMan chemistries are widely used to reliably genotype known and allele-specific polymorphic sites in a genome. TaqMan assays are robust in genotyping multiple variant types, including SNPs, small insertions/deletions (INDELs), and presence/absence variants [15]. However, TaqMan is expensive and less flexible in terms of assay design. KASP was developed as an alternative to TaqMan with the objective of reducing cost and improving genotyping efficiency, and it has now developed into a global benchmark technology [14]. More recently, a new genotyping platform called RNase H2 enzyme-based amplification (rhAmp) has been released [17, 18]. RNase H2 enzyme enables target-specific primer activation, which is followed by extension using a novel mutant Taq DNA polymerase that provides improved mismatch recognition [19]. rhAmp based on RNase H2-dependent PCR (rhPCR) combined with a universal reporter system attempts to reduce error rates from dimer formation and nonspecific amplifications while lowering costs compared with existing technologies.

Each system has its own merits and demerits. Some platforms are more comprehensive and relatively inexpensive, while others are highly precise for a specific purpose. An ideal genotyping platform should be flexible in assay design criteria, easy to run and cost-effective/affordable. So far, no single system fulfills all the requirements. The most competitive technologies in the current market are TaqMan, KASP and, as of recently, rhAmp. Therefore, this experiment was conducted to evaluate SNP genotyping performance of the three methods, TaqMan, KASP and rhAmp, in terms of assay design flexibility and success rates, allele calling and allele discriminating efficiency, ease of experiment run, and cost per sample.

## Materials and methods

### Plant materials and DNA extraction

Genomic DNA was extracted from 94 diverse wheat genotypes. DNeasy Plant Mini Kit (QIAGEN, United Kingdom) was used following the provider’s protocol. Agarose gel (0.6 g/100 ml) electrophoresis was used to compare light intensity of DNA samples with the known concentration (25, 50, and 100 ng/μl) of standard lambda DNA. DNA samples were normalized to 50 ng/μl for each sample. In addition to the 94 wheat samples, two non-template controls (NTCs) were included on each reaction plate.

### SNP assay design and genotyping

Assay probes were designed in the three platforms for 50 pre-validated SNPs or INDELs [20] that are closely linked to various genes controlling agronomic traits of wheat. Sequences flanking SNPs or INDELs were submitted for assay design to the respective genotyping platform providers (Thermo Fisher Scientific, LGC Genomics and Integrated DNA Technologies for TaqMan, KASP and rhAmp, respectively). Thirty-nine out of the 50 SNPs or INDELs passed assay design criteria across the three platforms. For the present study, 30 out of the 39 were selected for manufacturing genotyping assays across all the three technologies (S1 Table), and they were used for genotyping comparisons on 94 wheat lines.

Other genotyping reaction regents were purchased from the respective genotyping platform providers as well. TaqMan genotyping reactions were carried out in total volumes of 5 μl containing 1x TaqMan universal PCR master mix, 1x custom SNP genotyping assay mix and 5 ng DNA. KASP assay was also carried out in a 5 μl volume containing 1x KASP master mix, 1x custom KASP assay and 20 ng DNA. Similarly, rhAmp SNP genotyping was carried out in a 5 μl total volume containing 1x rhAmp genotyping master mix, 1x rhAmp reporter mix with reference dye and 5 ng genomic DNA. PCRs and fluorescent readings were taken in 384-well plates (Thermo Fisher Scientific) on QuantStudio 7 (QS7) Flex Real-Time PCR System (Applied Biosystems) following recommended thermal cycling conditions for each genotyping platform (Table 1). All genotyping experiments were conducted at the Integrated DNA Technologies (IDT) laboratory (Redwood City, California, USA). Thermo Fisher Cloud Genotyping application was used to automatically generate allele calls and allele discrimination plots for all three technologies.

**Table 1:** PCR protocols used for the three technologies.

Platform	PCR stage						
	Pre-read stage	Hold Stage 1	PCR stage 1		PCR stage 2		Post read stage
	Temp/time	Temp/time	Temp/time	cycles	Temp/time	cycles	Temp/time
TaqMan	60°C/30s	95°C/10m	95°C/15s 60°C/1m	40	-	-	60°C/30s
KASP	30°C/1m	94°C/15m	94°C/20s 61°C/1m	10	94°C/20s 55°C/1m	29	30°C/1m
rhAmp	60°C/30s	95°C/10m	95°C/10s 60°C/30s 68°C/20s	36	-	-	60°C/30s

### Statistical analysis

**Fig 1:**
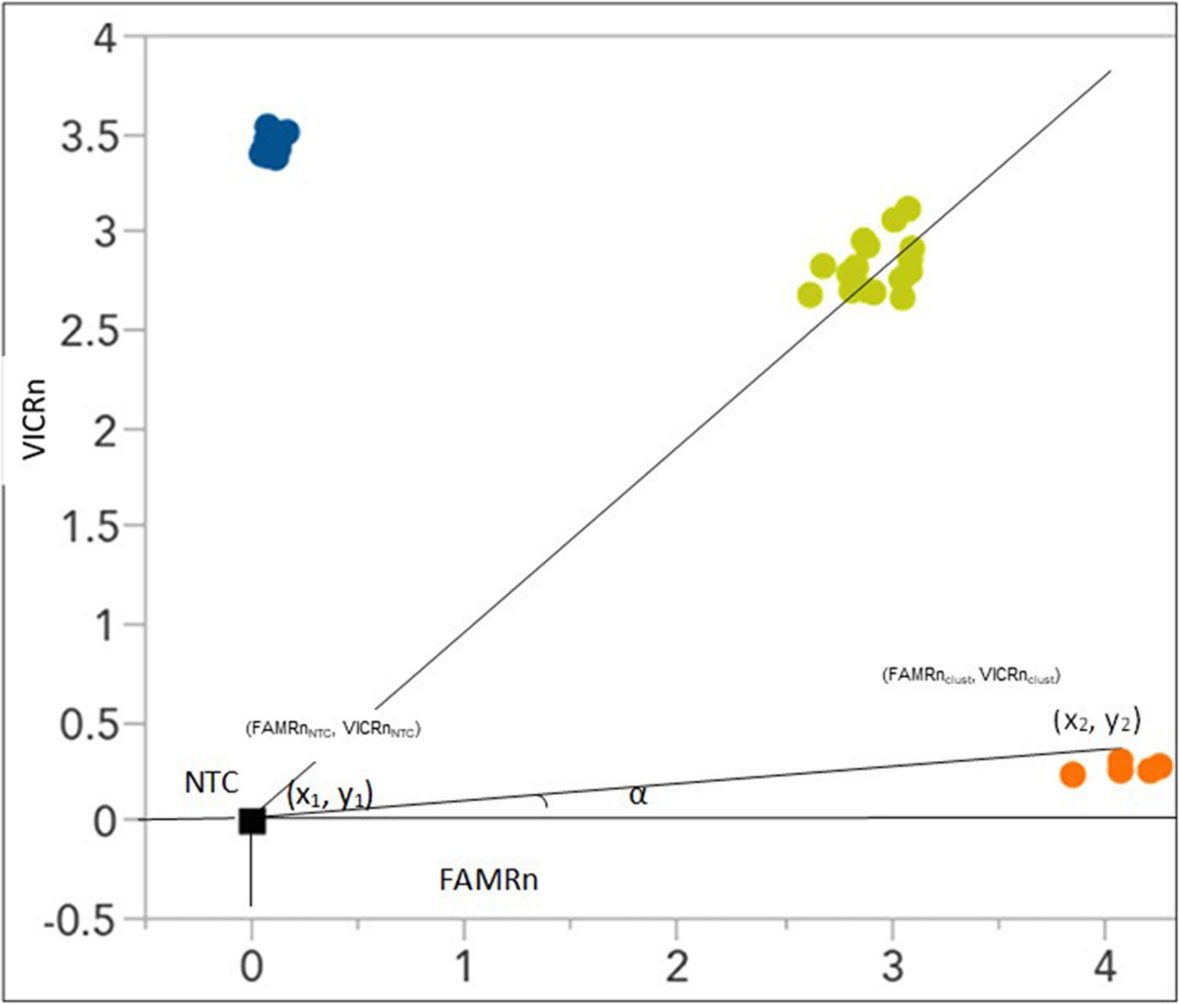
Schematic representation of graphical allele discrimination plot. The smaller the angle (α) between the horizontal and line connecting (x_1_, y_1_) and (x_2_, y_2_) the higher the discrimination efficiency of a platform, where x_1_ and y_1_ are the average fluorescence values of the no template control and x_2_ and y_2_ are the fluorescence values of each sample. The FAM and VIC fluorescence dyes used in this illustration only apply to TaqMan, other technologies used different florescence combinations.

Successful rate of assay design, allele call quality, allele discrimination (cluster separation angle), distance between allele clusters and non-template control (NTC) coordinates, and clusters compactness were compared among the three genotyping platforms. In an ideal case scenario of allele discrimination, homozygous allele 1 (Allele 11) lies along the horizontal axis (high FAM and low VIC signal in the case of TaqMan, for instance), while homozygous allele 2 (Allele 22) is expected to be parallel with the vertical axis (high VIC and low FAM signal). Heterozygote alleles (Allele 12) are expected to cluster along the diagonal (nearly equal FAM and VIC signals) (Fig 1). Using coordinate geometry principles, it is possible to calculate the angle size between the horizontal axis (X-axis) and the line connecting a data point (x_2_, y_2_) with the NTC coordinate (x_1_, y_1_). The slope of the line connecting data points with the NTC coordinate gives us the tangent of the angle α (Fig 1). Slope (tangent) = $\frac{\left(y_{2}-y_{1}\right)}{\left(x_{2}-x_{1}\right)}=\frac{\Delta y}{\Delta x}$. The inverse of the tangent was used to calculate the size of angle (α) separating each allele in a cluster in radians using the following formula: Angle of separation (α) = ${\text{tan}}^{-1}\frac{\Delta y}{\Delta x}$, where Δx is FAMRn_cluster_—FAMRn_NTC_ and Δy is VICRn_cluster_—VICRn_NTC_.

Another measure of an ideal case scenario of a good allelic discrimination is distance between allele clusters and average NTC data points. The distance between NTC and each data point was calculated using the formula D = $\sqrt{\Delta x^{2}+\Delta y^{2}}$ where, Δx is FAMRn_cluster_−FAMRn_NTC_ and Δy is VICRn_cluster_—VICRn_NTC_. Similarly, cluster compactness was evaluated as the standard deviation of the distances between data points in a cluster and average coordinate value of a cluster as follows: $S=\sqrt{\frac{\sum \left(x_{i}-\overset{-}{x}\right)}{n-1}}$, where x_i_ is distance of a data point from the average cluster coordinate, $\overset{-}{x}$ is the mean of distances of all data points in the cluster, and n is the number of data points in the cluster. The same distance formula was used to calculate the distance between each data point and the average coordinate of the cluster. Separation angle and cluster compactness were analyzed based on the values of Allele 11 for each platform. Call quality score is a function of the probability of the most likely base in a particular read based on the observed data. Analysis of variance (ANOVA) was conducted to evaluate statistical differences among the three technologies following completely randomized design (CRD) taking each successful amplification as a replication.

## Results

The three platforms did significantly differ in assay design success rate, allele call quality, allele cluster separation, cluster compactness and NTC-to-cluster distance (Table 2). Assay designs were started with 50 functional SNPs or INDELs of wheat across the three platforms. KASP and rhAmp both had higher successful design rates (49/50) than that of TaqMan (39/50) mainly because TaqMan probes cannot be designed if INDELs are more than six bps. Thus, only 30 sequences that contain target SNPs were used to design assays for all three platforms for genotyping platform comparisons (S1 Table). One SNP assay failed in all the three platforms. In each platform, 2,784 data points, including NTCs, were generated (96*29). Some samples failed to amplify even though the SNP assays worked well with other samples in the panel. Such failures may be ascribed to pipetting errors or primer mismatch due to mutations in these samples. As a result, TaqMan showed the highest number of unamplified samples (7%), followed by KASP (6.5%) and rhAmp (3%). Seventy-seven amplified samples had very low fluorescence making them difficult to be classified as either of the allele forms (Allele 11, 12 or 22). These alleles were reported as “Invalid” by allele auto calling using the Thermo Fisher Cloud Genotyping application. TaqMan showed 57 invalid allele calls, while KASP and rhAmp showed 13 and seven, respectively.

**Table 2:** Analysis of variance on allele call quality, allele discrimination (cluster separation angle), cluster compactness, and distance between NTCs and allele cluster coordinates.

Parameter	Source of variation	Degree of freedom	Sum squares	Mean squares	F	P
Call quality	Tech	2	0.24	0.12	27.9	8.463e-13
	Residuals	7624	32.9.4	0.004		
Separation angle*	Tech	2	0.31	0.16	6.07	0.004
	Residuals	59	1.54	0.03		
Cluster compactness*	Tech	2	0.46	0.23	35.08	1.17E-10
	Residuals	57	0.37	0.01		
NTC to cluster distance	Tech	2	182.02	91.01	294.3	2.20E-16
	Residuals	71	21.95	0.31		

*Cluster separation angle and cluster compactness were based on data values of Allele 11 for all three platforms.

Average allele call quality was generally high with 97% for rhAmp and 98% for KASP and TaqMan, respectively (Table 3). Genotype concordance between rhAmp and KASP was 86.10% while between KASP and TaqMan 82.17%, and rhAmp and TaqMan 90.36%, respectively.

**Table 3:** Mean separation of three genotyping platforms for allele call quality, allele discrimination (cluster separation angle), cluster compactness, and distance between NTC coordinate and allele clusters.

Platform	Allele call quality	Separation angle	Cluster compactness	NTC–cluster center distance
rhAmp	0.97^b^	0.19^b^	0.26^a^	5.01^a^
KASP	0.98^a^	0.19^b^	0.20^a^	1.51^c^
TaqMan	0.98^a^	0.35^a^	0.05^b^	1.93^b^

Significantly different values (LSD) were designated by different letters (a, b, c).

The angle between the X-axis and lines joining cluster points with the average NTC coordinate (FAMRn_NTC_, VICRn_NTC_) of the Cartesian plane (Fig 1) was taken as a measure of cluster separation. Allele 11 of each platform was used to compare cluster separation among the three platforms. ANOVA on the size of angles separating each data point from the horizontal showed highly significant (p < 0.001) variation among the three technologies (Table 2). rhAmp and KASP showed smaller angle compared to that of TaqMan, indicating better allelic discrimination between homozygous (Allele 11) and heterozygous (Allele 12) (Table 3). ANOVA showed that TaqMan had the most compact cluster, while rhAmp and KASP were not statistically different (Table 3).

**Fig 2:**
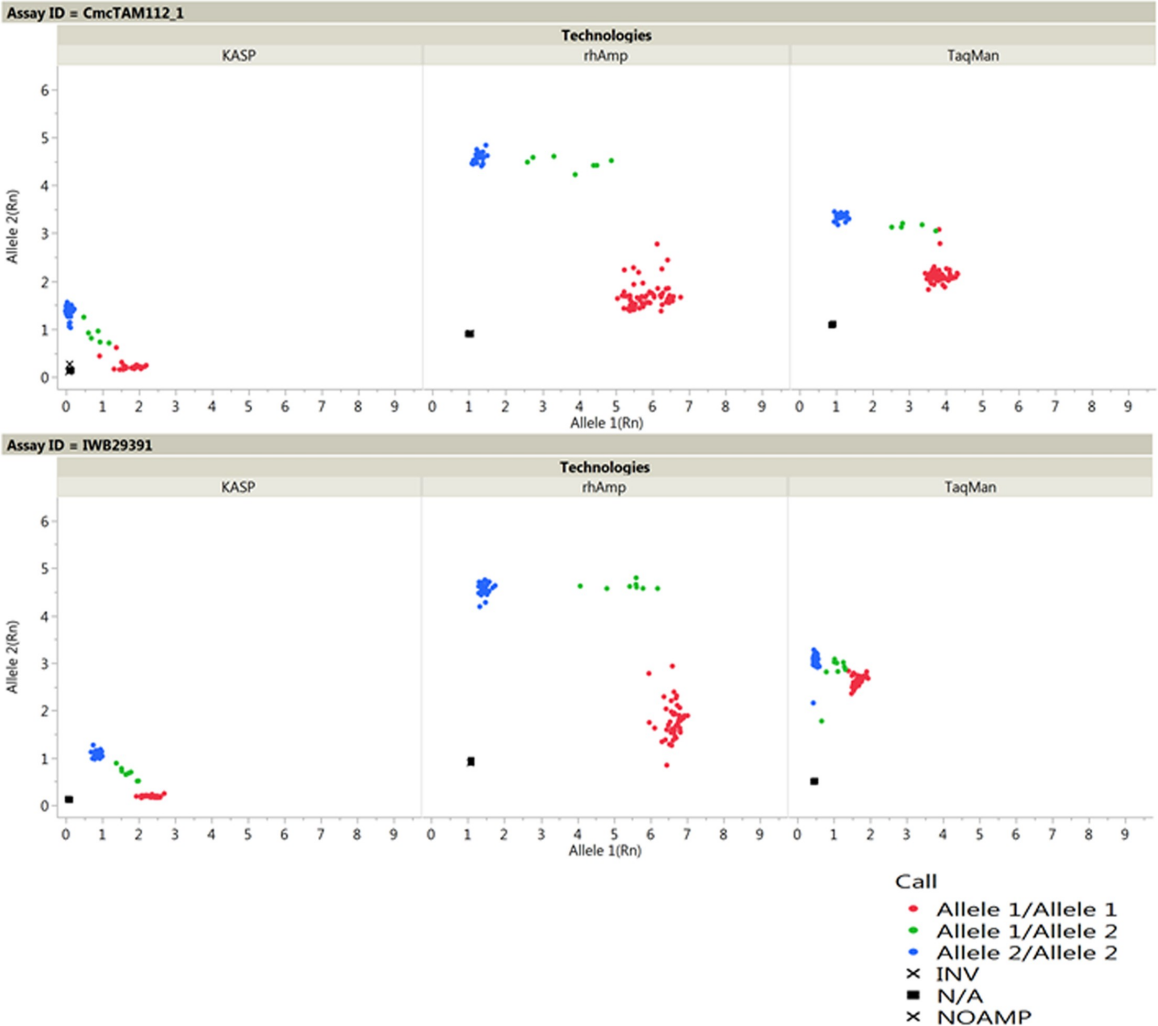
Example allele discrimination plots of the three genotyping platforms. All discrimination plots were drawn to similar scale to make them comparable. The X- and Y-axes indicate the fluorescence values of the dyes, while the dots are individual sample points.

The NTC to cluster distance is proportional to the amount of fluorescence of the two dyes. rhAmp showed the highest fluorescence, thereby showing the longest separation between NTC and cluster points, followed by KASP (Fig 2 and Table 3).

To calculate cost per sample, we considered the minimum amount of each kit (assay and master mix) one can order from the respective technologies and divided the total cost by the number of tests possible. rhAmp was the least expensive of the three technologies ($0.12 per 5 μl reaction), followed by KASP ($0.15 per 5 μl reaction), while TaqMan assays cost $0.41 per 5 μl reaction.

## Discussion

Small-scale high-throughput genotyping systems are in high demand in current plant breeding programs. TaqMan and KASP chemistries are among the most popular technologies in this regard so far. However, TaqMan is more expensive and less flexible in assay design because it does not accept INDELs longer than 6 bps. KASP requires higher amount of DNA template and yet its allele cluster separation is lower than that of TaqMan and rhAmp due to its low fluorescence signal. rhAmp was developed to fill these gaps at lower cost with more flexibility in assay design compared with TaqMan and better allele discrimination efficiency with less DNA input compared to KASP. Comparative performance of these three technologies in genotyping hexaploid species has never been reported before. This research was conducted to objectively compare the three technologies in terms of ease of assay design, allele call rate and quality, allele cluster compactness and angle of separation between allele clusters (homozygotes from heterozygotes).

Results in this study indicated that rhAmp was better in separating homozygous allele clusters from the heterozygotes (Table 3) which was in agreement with a similar study on sugar beet [18]. Compared with TaqMan and KASP technologies, rhAmp produced less ambiguous allele clusters. rhAmp technology uses blocked primers that help minimize primer-dimer formation and non-specific amplification [19]. rhAmp can be used as a routine genotyping platform for marker deployment and foreground and background selections in plant breeding.

The graphical view of allele clusters could be misleading in terms of the separation angle and cluster compactness, as the fluorescence of rhAmp was generally higher than that of the other two technologies. TaqMan had the most compact clusters due to the smaller projection of fluorescence away from NTC coordinate, while the opposite was true with rhAmp (Fig 2). Graphs needed to be drawn to scale based on the level of fluorescence of the two dyes. Because of its high level of fluorescence, rhAmp had a better sensitivity and confidence in identifying alleles with a low level of sample input.

In this study, rhAmp produced more amplified data points, only 3% failure compared with 7% and 6.5% unamplified samples in TaqMan and KASP, respectively. In a similar performance comparison between TaqMan and KASP, Braae and colleagues concluded that TaqMan showed higher failure rates than KASP [16]. In addition to the low design rate of TaqMan probes, many of them failed to amplify, which was in agreement with the previous study [16]. rhAmp is more sensitive in detecting rare variants because of its double enzyme mismatch detection and new mutant Taq DNA polymerase [19].

The three technologies varied in terms of the minimum amount of kit one needs to order and the number of tests possible in a given volume. The amount of assay was the limiting entity in the chemistry compared with the master mix in all three technologies. Price calculations were based on a 5 μl reaction volume for all technologies. TaqMan provided assay mix (primers and probes) enough for 2,000 reactions for $259 while KASP and rhAmp each provided assay mix enough for 5,000 reactions for $64.2 and $75.6, respectively. Similarly, KASP and rhAmp provided master mix of 25 ml at a cost of $1,382.5 and $1,038.6 enough for 10,000 reactions, respectively, while TaqMan provided 5 ml master mix enough for 2,000 reactions that cost $554. Cost estimations did not include price for DNA extraction. In addition to being more specific with high allele discrimination, rhAmp chemistry is highly affordable based on current market standards. rhAmp had the lowest cost per sample followed by KASP, while TaqMan was the most expensive.

Generally, all three technologies provide reliable allele calling for small-scale applications in plant breeding. However, the differences in allele cluster separation and distance of clusters from the NTC coupled with lower genotyping cost make rhAmp a preferred technology. TaqMan and rhAmp chemistries are real-time PCR technologies, which are amenable for real-time data collection or post-read technologies, while KASP is a post-read technology, which collects data after completion of the PCR process. In conclusion, rhAmp has the potential to be a viable alternative to both TaqMan and KASP technologies for small-scale SNP genotyping. Data generated in the present study will help make informed decisions regarding choosing efficient genotyping platforms. The assay design flexibility and allele discrimination clarity of rhAmp coupled with the availability of complete genome sequence of wheat [21] will enable the screening and validation of functional markers, further equipping the wheat breeding toolbox.

## Supporting information

S1 Table The 30 SNPs and their respective primer (and probe) sequences of the three genotyping platforms. (XLSX)
